# Establishment of a Preoperative Laboratory Panel to identify Lymph Node Metastasis in Superficial Esophageal Cancer

**DOI:** 10.7150/jca.71114

**Published:** 2022-04-11

**Authors:** Han Chen, Ruoyun Yang, Xin Yu, Xingzhou Jiang, Liuqin Jiang, Guoxin Zhang, Xiaoying Zhou

**Affiliations:** 1 Department of Gastroenterology, The First Affiliated Hospital of Nanjing Medical University, Nanjing 210029, China.; 2 The First Clinical Medical College of Nanjing Medical University, Nanjing 210029, China.

**Keywords:** Superficial Esophageal Cancer, Squamous Cell Carcinoma, Lymph Node Metastasis, Laboratory Panel, Decision tree

## Abstract

**Background and Aims:** In superficial esophageal squamous cell carcinoma (SESCC), the lymph node status is considered as one of the essential factors to determine the primary treatment strategy. Nevertheless, current noninvasive staging methods before surgical intervention have limited accuracy. This study aimed to establish a simple and noninvasive serum-testing panel that facilitates the preoperative prediction of pathological nodal status in SESCC patients.

**Methods:** Data for preoperative hematological parameters were retrospectively collected from 256 SESCC patients who underwent esophagectomy from December 2017 to May 2020. The random forest classification and decision tree algorithms were applied to identify the optimal combination of serum parameters for accurately identifying positive nodal metastasis.

**Results:** Twelve candidate parameters were identified for statistical significance in predicting positive nodal metastasis. A multi-analyte panel was established by using a random forest classification method, incorporating four optimal parameters: Hematocrit (HCT), Activated Partial Thromboplastin Time (APTT), Retinol-Binding Proteins (RBP), and Mean Platelet Volume (MPV). A schematic decision tree was yielded from the above panel with an 89.1% accuracy of classification capability.

**Conclusions:** This study established a simple laboratory panel in discerning the preoperative lymph nodal status of SESCC patients. With further validation, this panel may serve as a simple tool for clinicians to choose appropriate intervention (surgery versus endoscopic resection) for SESCC patients.

## Introduction

Superficial Esophageal Squamous Cell Carcinoma (SESCC) belongs to the early esophageal cancer. It is pathologically defined as the squamous cell carcinoma with mucosal (M) or submucosal (SM) invasion (Tis, T1a, and T1b), regardless of regional lymph node (LN) metastasis 1. Traditionally, esophagectomy is considered as the mainstay treatment, but this treatment has been reported to be associated with considerable postoperative morbidity and mortality during the past ten years 2. Endoscopic Submucosal Dissection (ESD) is an alternative treatment to esophagectomy. This minimally invasive procedure is absolutely-contradicted in those with suspicious lymph node metastasis before initial treatment 3. Thus, one's preoperative lymph node status is an essential factor for clinicians to determine the primary treatment strategy 4.

Principal modalities are usually considered as the noninvasive and radiography-based methods for assessing preoperative nodal status. These modalities include contrast-enhanced computed tomography (CT) scans, endoscopic ultrasonography (EUS), as well as fluorodeoxyglucose positron emission tomography (FDG-PET). Unfortunately, these methods have limited accuracy, which was reported to be 66% for EUS, 63% for CT, and 68% for PET, sensitivity 42%, 35%, and 35%, respectively, and specificity 91%, 93%, and 87%, respectively 5.

Recently, hematologic parameters have been frequently studied as diagnostic or prognostic indicators in many cancers. These parameters include inflammatory indices, coagulation factors, and some serum tumor biomarkers. For example, in resectable esophageal cancer, the lymph node metastasis is reported to be associated with the coagulation function where hypercoagulability and hyperfibrinolysis generally occur 6. Patients with other malignant tumors may also have increased inflammatory index or abnormal coagulation functions 7. However, the clinical significance of these parameters has not been studied in early esophageal cancer.

Thus, this study aims to investigate the clinical significance of preoperative serum parameters in predicting lymph node metastasis of early esophageal cancer. We also aim to establish a simple multi-analyte serum test facilitating the preoperative prediction of pathological nodal status in SESCC patients.

## Materials and Methods

### Patients and study design

Patients pathologically diagnosed with SESCC by endoscopic biopsies between December 2017 to May 2020 were consecutively identified by a clinical electronic database from a single center. A retrospective cohort was established, composed of 256 consecutive patients who underwent primary surgical resection for SESCC. Inclusion criteria were listed as follows: (1) histopathological diagnosis of esophageal squamous cell carcinoma on surgical specimens; (2) pT1 stage carcinoma (no tumor invasion beyond the submucosa); (3) primary surgical resection with a three-field lymphadenectomy; (4) no history of previous malignancies and anticancer therapies. Exclusion criteria: (1) histopathological diagnosis of esophageal adenocarcinoma or other types of esophageal cancer; (2) mixed type of esophageal cancer; (3) tumor with the undefined pathological origin and metastatic esophageal cancer; (4) esophagostomy after endoscopically resection; (5) patients younger than 18 years; (6) perioperative mortality; (7) distant metastases; (8) previous medical history of hematologic or rheumatic autoimmune disease; (9) acute or chronic infections during inpatient stays; (10) a previous history of taking aspirin or warfarin. The study was approved by the Institutional Ethics Committee.

### Data collecting procedure

The whole blood specimens were collected from each patient before surgery. The Beckman Coulter UniCel® DxH 800 was used for analyzing routine blood markers including White Blood Cell (WBC), Neutrophils, platelet (PLT), Red Blood Cell (RBC), Hemoglobin; hematocrit (HCT), Platelet Distribution Width (PDW), Mean Platelet Volume (MPV). Inflammatory markers of Neutrophil-Lymphocyte Ratio (NLR), Platelet-Lymphocyte Ratio (PLR), and Monocyte-to-Lymphocyte Ratio (MLR) were calculated subsequently. The Sysmex® CS-5100 hemostasis system was applied for coagulation analysis of fibrin (FIB), Activated Partial Thromboplastin Time (APTT), Thrombin Time (TT), Prothrombin Time (PT), and D-dimer. The Beckman Coulter AU5800 Clinical Chemistry Analyzer was used for assessing lactate dehydrogenase (LDH) and retinol-binding proteins (RBP). The Roche® Cobas e602 module was used for tumor markers of Alpha-Fetoprotein (AFP), Carcinoembryonic Antigen (CEA), Cancer antigen 19-9 (CA199), cytokeratin 19 fragment (cyfra21-1), and Neuron Specific Enolase (NSE).

Besides, for comparing with the radiographic predicting method, results of contrast-enhanced computed tomography (CT) scans were recorded. Positive radiographic results were defined as an enlarged lymph node with a short axis dimension ≥1m in CT.

The three-field lymphadenectomy was conducted during surgery: in the cervix, supraclavicular and paracervical esophageal LNs are dissected; in the thoracic, paraesophageal LN, paratracheal LN, posterior mediastinal LN, supradiaphragmatic LN, and LNs around the bilateral recurrent laryngeal nerve are routinely dissected; in the abdominal, paracardial LNs, inphradiaphragmatic LNs, LNs along the lesser curvature, LNs along the trunk of the left gastric artery, and LNs around the abdominal esophagus (No. 20), and are dissected. The pathologic diagnosis was performed by two experienced pathologists independently. The 8th edition AJCC/UICC staging system of esophageal cancer was applied in all patients 8. The tumor size was measured in two dimensions as the maximum diameter by Vernier's calipers. Location (L) is defined by the position of epicenter of tumor. If no statement of epicenter is provided, the following measurements would be applied: (1) upper: 15-24cm from incisors; (2) middle: 25-29cm from incisors; (3) low: 30-40/45cm from incisors. Histologic grade (G) was categorized as well-differentiated (G1), moderately differentiated (G2) and poorly differentiated (G3) 21. Macroscopic tumor type was classified by using the 2016 Japanese Classification of Esophageal Cancer, 11^th^ Edition: Superficial type, protruding type, ulcerative and localized type, ulcerative and infiltrative type 9. The invasion depth was divided into four categories: epithelium (EP)/lamina propria mucosa (LPM), muscularis mucosa (MM), submucosal (SM)1, SM2 or deeper. Invasion depth and LVI was further confirmed by immunohistochemical staining.

### Statistical Analysis

Significant predictors for nodal prediction were identified by IBM SPSS Statistics for Windows, Version 23.0 (SPSS, Chicago, IL). Descriptive statistics and graphical displays were obtained for the range of concentration values associated with each marker. Data out of the upper or lower limits of each index was disqualified for further analysis. Normality tests were applied by Shapiro-Wilk and Kolmogorov-Smirnov test. An unpaired, two-tailed t-test was used for continuous parameters with normal distribution, and a nonparametric Mann-Whitney U-test for data with the abnormal distribution. Variables significantly associated with nodal status (p≤0.2) were identified as candidate predictors for multivariate analysis. These results were then integrated into the R software (version 3.5.1, http://www.R-project.org/) to formulate classifications by using the “randomForest” package 10. This package was responsible for identifying the optimal classification of investigated variables by creating multiple decision trees for each potentially powerful panel. Each tree was created (without pruning) through a cross-validation test where a training cohort (70% of the data) is randomly selected from the full study samples. This tree was used to predict the group membership for the remaining data, which is termed as an out-of-bag (OOB) prediction. This process is then repeated 500 times with generating a new training cohort each time and a new decision tree is created and used to perform a new OOB prediction. After a large number of trees have been grown, each tree voted for the most popular class. The OOB error rate was used to measure the classification accuracy of the random forest. This cross-validation is also used to compute a list of variables ordered by their importance scores generated by the randomForest package. The Gini importance of a variable is computed as the normalized total reduction of the criterion brought by that variable. The stepwise selection method sequentially searches for optimal subpanel where a variable had the lowest variable Gini importance score was removed from the forest at each step 11.

After identifying the optimal panel for predicting a patient's nodal status, the 'Rpart' package was used for drawing an optimal decision tree by implementing the Classification and Regression Tree (CART) algorithm. This tree was split into different branches by variables through a set of binary if-then logical (split) conditions that permit accurate classification of one's nodal status. The 'goodness of split criterion' is used to determine the best split point for each variable. After ranking all of the “best” splits, CART assigns classes to the two split nodes by following a rule of minimizing mistakenly-classified error. This process is persistent until all samples were perfectly classified. The final output of the resulting classification tree is a graphical display of decision criteria for each split, with the resulting predicted group memberships at the terminal nodes 12.

The optimal cutoff value of the tree was assessed by the Youden index in the receiver operating characteristic (ROC) curve. The area under the receiver operating characteristic curves (AUC) were calculated to evaluate the diagnostic accuracy. The ROC curves were plotted using the “pROC” package. The reported statistical significance levels were two-sided and statistical significance was considered as *p* ≤ .05.

## Results

### Significant individual serum markers for nodal prediction

Table 1 summarized the baseline characteristic of the study group. 256 patients (194 males and 62 females; median age, 61.2 years old; range, 36-83 years old) underwent surgical treatment. The overall incidence of lymph node metastases was 26.5% (68/256). Table 2 showed the information of patients with positive Lymph Node Metastasis. The Serum concentrations of RBP were found to be significantly higher in the nodal positive group (Mann-Whitney two-sided *p*<0.001) whereas the concentration of RBC, HGB, HCT, MPV, PT, APTT, were significantly lower in the nodal positive group (*p*-values 0.045, 0.045, <0.001, <0.001, 0.049 and <0.001, respectively). The difference in LY, CEA, NLR, FIB, and D-Dimer concentration between the nodal negative and positive groups was marginally significant, with a *p*-value of 0.091, 0.071, 0.123, 0.149, and 0.173, respectively. These above twelve serum parameters were individually identified to classify patients based on lymph node status. The RBP, RBC, HGB, HCT, MPV, PT, APTT, LY, CEA, NLR, FIB, and D-Dimer exhibited the most promising overall profiles. The results are presented in Table 3. We further classified the invasion depth into four categories: epithelium (EP), lamina propria mucosa (LPM), muscularis mucosa (MM), submucosal (SM)1, SM2 or deeper. However, no statistically significant correlation between HCT/APTT/RBP/MPV and invasion layers was found. These results were shown in Supplementary Table 1.

### Establishing a Laboratory Panel

**Figure 1:**
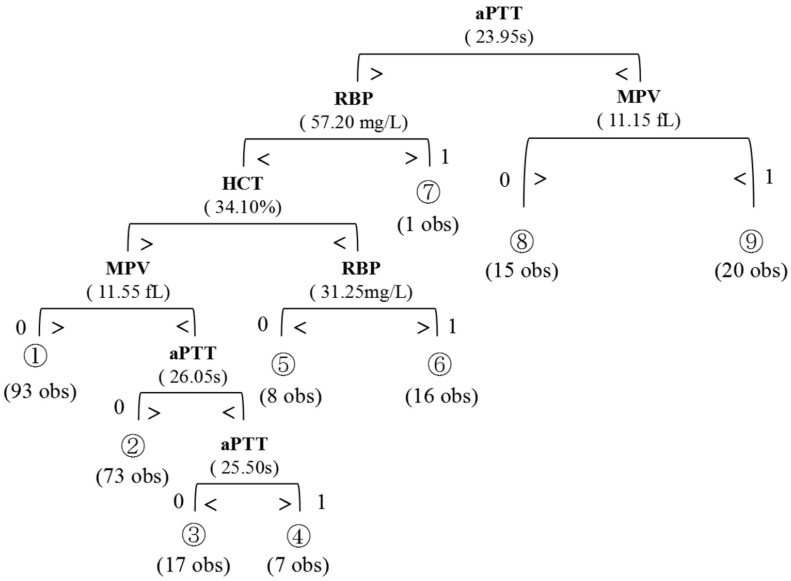
Regression Tree used for the prediction of preoperative lymph node metastasis in SESCC patients. A binary algorithm is applied to classify the parameters by referring to the cutoff in each separate tree branches. The number of classifications are shown below each terminal node (0, node negative; 1, node positive).

**Figure 2:**
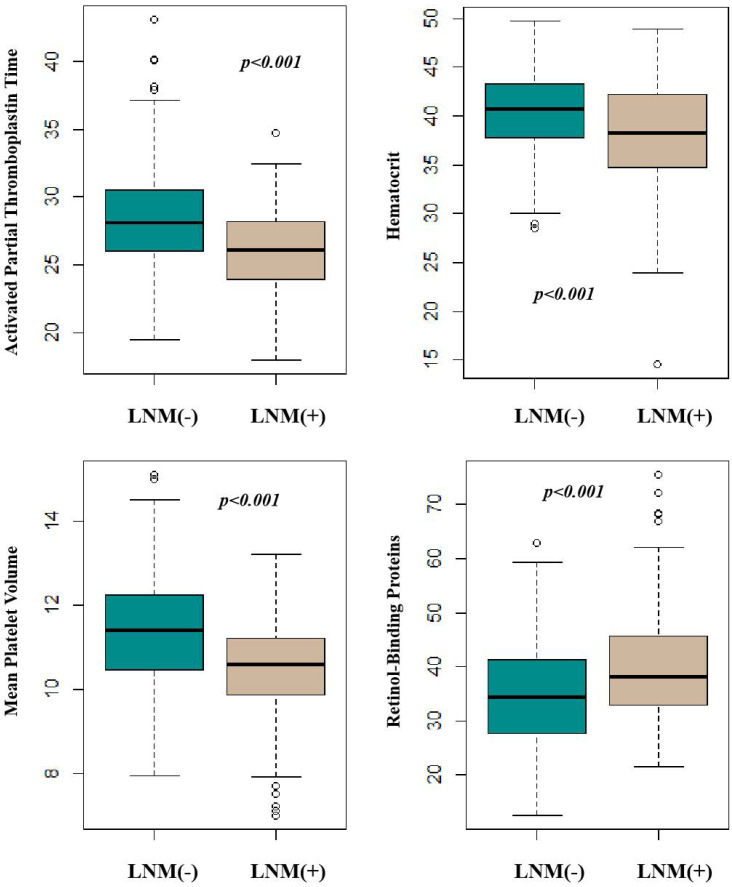
Comparison between statistically-significant serum parameters according to lymph nodal status.

With the CART methodology, a classification tree incorporating four serum markers (RBP, MPV, HCT, APTT) was applied to finally predict the preoperative nodal status. The final output of the resulting tree is displayed with the decision criteria for each split and the predicted results at the terminal nodes (Figure 1). The four markers from this panel were selected from the 12 candidates by the Random Forests algorithm. The comparison between HCT/APTT/RBP/MPV and lymph nodal status were shown in Figure 2. Table 4 presents the Gini importance and different combinations of each candidate variable. Clearly, RBP, MPV, HCT, APTT had higher Gini importance than the other eight variables, indicating that they weighted more in predicting preoperative nodal status than other candidates. This combination had the lowest OBB rate (10.94%) than others. The classification tree achieved an accuracy of 89.1% (228/256). AUC was calculated to be 0.89, with 66.2% sensitivity, 97.3% specificity, 90.0% PPV, and 88.3% NPV. There were 28 cases in which the nodal status was erroneously classified by this panel. The nodal status was underestimated in 23 patients (predicted negative but had positive pathological nodal status), and was overestimated in 5 patients (predicted positive but had negative pathological nodal status).

**Figure 3:**
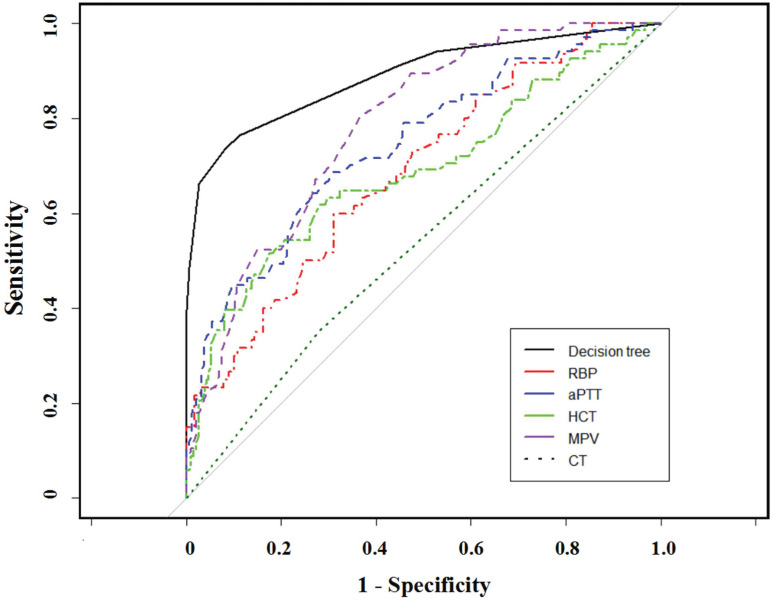
Comparison of ROC curves for different staging parameters by using Random Forest algorithms.

In contrast, the CT-based radiographic result presented with a 53.9% accuracy rate, 20.6% sensitivity, and specificity of 66.0%. CT-based stage discrepancies consisted of 54 underestimated and 64 overestimated patients upon pathologic staging, the error rate of which is far more than the serum panel method. The lymph status of 20 patients was underestimated by both methods, while only 2 patients were overestimated. Figure 3 showed the compared AUC in the established model, CT scans, and other single parameters. This laboratory panel presented increased AUCs than other methods, with all *p*<0.05.

## Discussion

Our results confirm that several serum markers are statistically significant in predicting the preoperative lymph nodal status of SESCC. Since the lymph node status plays an essential role in determining whether endoscopic resection can be performed as the primary treatment for SESCC, the application of our model help identify patients with low risks of nodal metastasis before surgery. These low-risk patients may choose endoscopic resection and avoid unnecessary surgical operations. Also, clinicians can use this serum-testing panel to monitor the metastatic risks of lymph nodes because these four parameters were less invasive and easily accessed during clinical practice. To our knowledge, this is the first serum-testing panel served as a clinical tool to predict one's lymph node status in SESCC. This panel incorporated the four most significant hematologic markers, including RBP, MPV, HCT, and aPTT, which are significantly associated with lymph node metastasis in SESCC.

These four markers have all been investigated separately in previous researches. Our finding of shortened aPTT in patients with positive lymph node metastasis is consistent with the findings of two retrospective studies conducted by Liu et al. 13 and Li et al. 14, respectively. Although they did not study cancer from the esophagus, they both confirmed that aPTT may be an indicator of positive nodal status in non-small cell lung cancer and oral squamous cell carcinoma, as thrombophilia may potentially occur during the process. MPV is a marker that reflects platelet activity. Ohuchi et al. reported that decreased MPV is associated with lymph node metastasis and poor overall survival in lung cancer patients 15. Likewise, in our study, the serum level of MPV is lower in SESCC patients with positive nodal status, which may be explained by the association between coagulation and cancer status. In general, there is an inverse relationship between the platelet count and the MPV 16. Tumor cells may release some soluble proinflammatory and proangiogenic factors, which stimulates the prothrombotic properties of vascular cells. Subsequently, large platelets tend to react to these stimuli. This process may cause selective consumption of platelets, and the serum concentration of MPV of circulating platelets is decreased accordingly 17. HCT means the proportion of blood volume by red blood cells. One study conducted by Lin et al 18 investigated the clinical significance of HCT in Gastric Cancer Patients. They found that the low HCT may be associated with metastatic potential. In our study, the decreased HCT is also identified in SESCC patients with positive nodal status. The possible explanation is that the lower level of HCT indicates the reduced oxygen-carrying capacity 19. Under this condition, hypoxia may occur, which may increase the metastatic potential by inducing proteomic and genomic changes 20. No previous studies directly investigated the relationship between RBP and esophageal cancer. Bichler et al. reported that the plasma levels of RBP in patients with squamous cell carcinomas of the head and neck were significantly lower than in tumor-free individuals 21. High circulating levels of RBP was found to be associated with increased breast cancer risk 22. Interestingly, in our study, there is a slightly increased concentration of plasma RBP in SESCC patients with positive LN metastasis than in node-negative ones. As RBP was reported to be a tumor marker 23, further studies are needed to investigate the potential mechanism of RBP in the progression of positive nodal metastasis in SESCC.

Our 4-analyte panel accurately predicted the lymph node metastasis in 89.1% of the SESCC patients tested. Traditionally, CT is one of the most frequently used preoperative staging methods by measuring the size of regional lymph nodes. However, the clinical stage of the lymph node cannot be determined by lymph node size alone. False-negative results are frequently seen in normal-sized nodes that contain metastatic deposits, while false-positive examinations are seen in non-malignant nodal enlargement, such as inflammatory nodes. Van et al reported in a review that CT was 50% sensitive and 83% specific in preoperative assessment of lymph node metastasis in esophageal cancer 24. Our study found that the sensitivity and specificity of CT scan were lower in predicting nodal status in superficial esophageal cancer. The possible reason is that the size change of the lymph node is not significant in the early stage of esophageal cancer. Compared with CT, our serum panel presented a better diagnostic evaluation in accuracy, sensitivity, specificity, and AUC.

Our model is the first decision tree model in SESCC established for lymph node prediction. This model works on the concept of information gain at every node. It then classifies data points at each of the nodes and checks for information gain at each node. A decision tree is a simple and easy-to-understand model. By using this model, clinicians can directly evaluate the preoperative nodal status and further consider the treatment strategy. However, the main limitation of this model is the possible over-fitting of data with multiparametric statistical models 25, 26. To decrease the possibility of over-fitting, we conducted cross-validation across several multiparametric models. Besides, we also calculated the OBB rate for every combination and finally chose the optimal combination with the lowest OBB. Another limitation is that our model was developed from a single-center retrospective cohort. Thus, the external validation outside our hospital is further needed to test model performance. We expect more improved machine learning models that can be established by a complicated deep learning algorithm in the future.

## Conclusion

We established a simple laboratory panel in discerning the preoperative nodal status of SESCC patients. With further validation, this panel may serve as a simple tool for clinicians to choose appropriate intervention for SESCC patients. We predict this insight will allow us to more effectively treat patients with SESCC and thereby improve life quality and decrease postoperative complications.

## Supplementary Material

Supplementary table.

## Figures and Tables

**Table 1:** Patient demographics of patients with superficial esophageal cancer

Variables	Patients with Superficial Esophageal Cancer (n=256)
**Sex**	
Male, n (%)	194 (75.8%)
Female, n (%)	62 (24.2%)
**Age (year)**	
Median, Range	63 (42-77)
Tumor Size (cm)	
Median, Range	2.0 (0.3-11.0)
**SESCC Stage, n (%)**	
**cTNM**	
IA/IB	230 (89.8%)
IIA/IIB	0 (0.0%)
IIIA/IIIB	0 (0.0%)
IVA	26 (10.2%)
IVB	0 (0.0%)
**pTNM**	
IA	63 (24.6%)
IB	125 (48.8)
IIA	0 (0.0%)
IIB	53 (20.7%)
IIIA	12 (4.7%)
IIIB	0 (0.0%)
IVA	3 (1.2%)
IVB	0 (0.0%)
**Tumor location, n (%)**	
Upper thoracic	43 (16.8%)
Middle thoracic	97 (37.9%)
Lower thoracic	116 (45.3%)
**Histologic Grade, n (%)**	
Well	25 (9.7%)
Moderately	119 (46.5%)
Poorly	112 (43.8%)
**Invasion Depth, n (%)**	
Mucosal layer	70 (27.3%)
Submucosal layer	186 (72.7%)

**Note:** Clinicopathological characteristics of the tested patient population. SESCC, superficial esophageal squamous cell carcinoma. SESCC Stage* includes both clincial (cTNM) and pathological stage (pTNM) based on the 8^th^ edition AJCC/UICC staging system of esophageal cancer.

**Table 2:** The clinicopathological characteristics of patients with positive lymph node metastasis

Variables	Patients with positive LN metastasis (n=68)
**Sex**	
Male	41 (39.7%)
Female	27 (60.3%)
**Age**	
≥60	40 (58.5%)
<60	28 (41.2%)
**Tumor Location**	
Upper thoracic	9 (13.2%)
Middle thoracic	24 (35.3%)
Lower thoracic	35 (51.5%)
**Tumor Size**	
≥ 3cm	17 (25%)
< 3cm	51 (75%)
**Histological grade**	
Well-differentiated	4 (5.9%)
Moderately-differentiated	35 (51.5%)
Poorly-differentiated	29 (42.6%)
**Invasion Depth**	
EP/LPM	1 (1.5%)
MM	5 (7.4%)
SM1	27 (40.0%)
SM2 or deeper	35 (51.5%)
**Lymphovascular Invasion**	
Positive	7 (10.3%)
Negative	61 (89.7%)
**Pathologic type**	
Protruding	16 (23.5%)
Superficial type	34 (50.0%)
Ulcerative and localized	15 (22.1%)
Infiltrative	3 (4.4%)
**Location of metastatic LN**	
Cervical	21 (30.9%)
Mediastinal	14 (20.6%)
Abdominal	26 (38.2%)
≥2 of the above areas	7 (10.3%)

**Table 3:** Individual serum markers in classifying nodal status in SESCC

Serum markers	LN metastasis* (+), n=68		LN metastasis* (-), n=188		Mann-Whitney U
	Median	Range	Median	Range	
aPTT (s)	25.60	18.00-34.70	28.15	19.50-43.10	<0.001
MPV (fL)	10.30	7.00-12.60	11.60	7.93-15.10	<0.001
HCT (%)	37.00	14.60-48.90	40.65	28.50-49.70	<0.001
RBP (mg/L)	38.65	25.10-75.50	34.00	12.60-55.80	<0.001
FIB (g/L)	2.74	1.31-25.20	2.54	0.17-6.46	0.149
CEA (ng/L)	2.10	0.40-9.20	2.46	0.59-29.41	0.071
LY (10^^9^/L)	1.65	0.56-5.31	1.51	0.23-3.15	0.091
NLR	1.95	0.09-10.58	2.09	0.42-28.30	0.123
RBC (10^^12^/L)	4.21	2.36-5.38	34.00	12.60-55.80	0.045
PT (s)	11.55	8.70-13.80	11.70	10.40-18.90	0.049
DD2 (mg/L)	0.28	0.10-6.46	0.25	0.04-7.96	0.173
HGB (g/L)	129.50	78.00-176.00	135.00	63.00-169.00	0.045

**Note:** *Based on pathologic staging. RBC, Red Blood Cell; Hemoglobin; HCT, Hematocrit; MPV, Mean Platelet Volume; NLR, Neutrophil-Lymphocyte Ratio; FIB, Fibrin; PT, Prothrombin Time; aPTT, activated Partial Thromboplastin Time; DD2, D-dimer; RBP, retinol-binding proteins; CEA, Carcinoembryonic Antigen.

**Table 4:** Variable selection of the 12 serum marker candidates

Variables	Gini importance	Combinations of laboratory parameters													
		C1	C2	C3	C4	C5	C6	C7		C8		C9	C10	C11	C12
aPTT (s)	19.59	+	+	+	+	+	+	+	+		+		+	+	+
MPV (fL)	15.67	+	+	+	+	+	+	+	+		+		+	+	
HCT (%)	13.67	+	+	+	+	+	+	+	+		+		+		
RBP (mg/L)	11.33	+	+	+	+	+	+	+	+		+				
FIB (g/L)	6.83	+	+	+	+	+	+	+	+						
CEA (ng/L)	5.93	+	+	+	+	+	+	+							
LY (10^^9^/L)	5.55	+	+	+	+	+	+								
NLR	5.24	+	+	+	+	+									
RBC (10^^12^/L)	4.45	+	+	+	+										
PT (s)	4.15	+	+	+											
DD2 (mg/L)	4.14	+	+												
HGB (g/L)	3.33	+													
**OBB Error Rate (%)**		14.79	17.12	16.73	16.34	15.95	15.18	13.62	13.23		10.94		15.56	22.96	27.24
															

**Note:** Gini importance represents the importance of the single index on the left side of the table in predicting Lymph node metastasis. OBB Error Rate (%) was calculated from different combinations of parameters. + Represents the single index on the left side was involved in the parameter combination in the longitudinal direction. Abbreviates: RBC, Red Blood Cell; Hemoglobin; HCT, Hematocrit; MPV, Mean Platelet Volume; NLR, Neutrophil-Lymphocyte Ratio; FIB, Fibrin; PT, Prothrombin Time; aPTT, activated Partial Thromboplastin Time; DD2, D-dimer; RBP, retinol-binding proteins; CEA, Carcinoembryonic Antigen. OOB, out-of-bag.
