# Supplementary material for: Establishment of a Preoperative Laboratory Panel to identify Lymph Node Metastasis in Superficial Esophageal Cancer
Source: J Cancer. 2022 Apr 11;13(7):2238–45. doi: 10.7150/jca.71114 (PMC9066211; doi:10.7150/jca.71114)
Supplement: Supplementary file 1 — Supplementary table. [file jcav13p2238s1.pdf]

Supplementary Table 1. Correlation analysis between HCT/APTT/RBP/MPV and invasion layers/numbers of positive metastatic lymph nodes

	Correlation Coefficient			
Variables	Tumor invasion layers	p	Number of positive metastatic lymph nodes	p
aPTT	0.263	0.057	0.122	0.441
MPV	-0.042	0.734	-0.171	0.209
HCT	-0.105	0.396	0.174	0.195
RBP	-0.007	0.960	-0.057	0.690

Notes: Invasion layers were further ordered into four ranks: epithelium (EP)/lamina propria mucosa (LPM), muscularis mucosa (MM), submucosal (SM)1, SM2 or deeper. Correlation analysis was performed by using a Spearman's Rank Order Correlation. Correlation Coefficient and p-value were recorded. HCT, Hematocrit; MPV, Mean Platelet Volume; aPTT, activated Partial Thromboplastin Time; DD2, D-dimer; RBP, retinol-binding proteins; CEA, Carcinoembryonic Antigen.
